# MecVax, an Epitope- and Structure-Based Broadly Protective Subunit Vaccine Against Enterotoxigenic *Escherichia coli* (ETEC)

**DOI:** 10.3390/microorganisms13122866

**Published:** 2025-12-17

**Authors:** Weiping Zhang

**Affiliations:** Department of Pathobiology, University of Illinois Urbana-Champaign, 2001 South Lincoln Avenue, Urbana, IL 61802, USA; wpzhang@illinois.edu; Tel.: +1-217-300-7307

**Keywords:** ETEC, vaccine, MecVax, diarrhea, MEFA, adhesin, toxoid, platform

## Abstract

No vaccines are licensed against enterotoxigenic *Escherichia coli* (ETEC), a leading diarrheal cause in children and travelers. ETEC adhesins and enterotoxins are the virulence determinants and become the primary targets in ETEC vaccine development. However, ETEC strains produce > 25 adhesins and two potent enterotoxins, particularly the poorly immunogenic heat-stable toxin (STa), greatly hindering ETEC vaccine development. To overcome these challenges, we developed a multiepitope-fusion-antigen (MEFA) platform. MEFA presented multiple adhesin epitopes on a backbone and generated a polyvalent adhesin immunogen, CFA/I/II/IV MEF. CFA/I/II/IV protected against the seven ETEC adhesins (CFA/I, CS1-CS6) associated with two-thirds of ETEC diarrheal cases. We further used toxoids as safe antigens and created a toxoid fusion, 3xSTa_N12S_-mnLT_R192G/L211A_. This antigen induced antibodies neutralizing the enterotoxicity of STa and heat-labile toxin (LT), which, alone or together, cause all ETEC diarrheal cases. By combining two polyvalent proteins, we developed a multivalent ETEC vaccine, MecVax, that protects against seven ETEC adhesins and two enterotoxins. MecVax is broadly immunogenic. MecVax prevents intestinal colonization by ETEC strains expressing any of the seven adhesins and protects against clinical diarrhea from ETEC strains producing LT or STa enterotoxin preclinically, becoming a broadly protective ETEC vaccine candidate against children’s diarrhea and travelers’ diarrhea.

## 1. Introduction

Enterotoxigenic *Escherichia coli* (ETEC) strains produce adhesins that bind host receptors to colonize the small intestine and deliver enterotoxins that disrupt intestinal epithelial cell homeostasis. ETEC strains are the primary cause of diarrheal disease in animals and humans, particularly in young animals and children [1,2]. Indeed, ETEC infection is among the top four causes of diarrhea in children under 5 years old in developing countries [3,4], and it is also a common cause of diarrhea in the elderly population [5,6]. Additionally, ETEC is often the most common cause of diarrhea among travelers, particularly those from high-income countries traveling to ETEC-endemic regions or countries, including civilian and military personnel deployed in these areas [7,8,9]. ETEC infection is estimated to be associated with more than 200,000,000 clinical diarrhea cases and up to 80,000 deaths annually [10,11,12]. ETEC diarrhea is further linked to long-term negative impacts in children, including poor physical growth and impaired cognitive development [13,14,15,16]. Currently, there are no effective countermeasures against ETEC diarrhea [17,18,19].

ETEC diarrhea, like other enteric infections, is preventable. Clean drinking water and improved sanitation and hygiene (WASH) would effectively prevent ETEC infection, as demonstrated in industrialized countries where ETEC infection is under control. Unfortunately, due to financial constraints, rapid implementation of nation- or community-wide sanitation systems and improvements to water supply infrastructure, which require substantial investment, are unlikely to be achieved in the coming decades in developing countries, particularly in ETEC-endemic countries in South and Southeast Asia, Sub-Saharan Africa, and Central America. Medical interventions, including the use of antibiotics and oral rehydration with a salt-and-electrolyte solution, can effectively treat severe ETEC diarrheal cases. However, commonly prescribed antibiotics are less effective, or even ineffective, due to the increasing prevalence of antimicrobial resistance (AMR) in ETEC bacteria [20,21,22,23,24]. A lack of medical facilities in rural areas limits access to rehydration treatment. Vaccines administered to a large population to build herd immunity and prevent disease transmission would be a highly effective countermeasure against ETEC diarrhea and other infectious diseases. Sadly, there are no vaccines licensed against ETEC children’s diarrhea and travelers’ diarrhea.

Developing effective vaccines against ETEC diarrhea has historically been challenging. The key difficulties include (1) virulence heterogeneity among ETEC strains, (2) difficulties in protecting against ETEC enterotoxins, and (3) a lack of suitable animal models for vaccine preclinical efficacy assessment. ETEC bacterial strains produce two types of virulence determinants: adhesin and enterotoxin. Adhesins include colonization factor antigens (CFAs) and coli surface antigens (CSs). These adhesins promote ETEC bacterial attachment to host receptors and colonization of the host small intestine. Enterotoxins include heat-labile toxin (LT) and heat-stable toxin (STa). These enterotoxins enter host intestinal epithelial cells and significantly elevate intracellular levels of cyclic adenosine monophosphate (cAMP) or guanosine monophosphate (cGMP). An elevation of cAMP or cGMP disrupts host cell homeostasis and causes fluid hypersecretion into the gut lumen, resulting in watery diarrhea. The problems are that ETEC strains produce more than 25 immunologically heterogeneous adhesins and two potent and very distinctive enterotoxins. Since an ETEC strain that produces any one or two adhesins and one toxin can cause diarrhea, an effective ETEC vaccine needs to induce broad immunity to protect against many (if not all) ETEC adhesins and both toxins. Unfortunately, developing a vaccine against these many ETEC adhesins and two toxins has been overwhelmingly difficult [19]. Second, LT and STa are potent toxins; therefore, they are unsafe to be used directly as vaccine antigens. Moreover, the potent 19-amino acid STa toxin is poorly immunogenic. It has long been believed that an STa antigen can induce neutralizing antibodies only when it retains its enterotoxicity (which cannot be a safe antigen for a vaccine), but not from any nontoxic STa mutants [25]. Therefore, STa has generally been excluded as an ETEC vaccine antigen for decades. The dilemma is that STa plays a more important role in causing children’s diarrhea and travelers’ diarrhea [26,27]; thus, safe STa antigen(s) need to be carried by an effective ETEC vaccine to induce protective anti-STa immunity [19,28,29,30,31]. Third, there is not a single suitable animal model available to evaluate the preclinical efficacy of ETEC vaccine candidates. Mice, including zinc-deficient mice [32], cannot be efficiently colonized by ETEC bacteria in the small intestines and are not naturally susceptible to ETEC infection. Rabbits can be colonized by ETEC strains [33,34], but they rarely develop clinical diarrhea after ETEC infection. While pigs [35,36] and nonhuman primates [37,38] are susceptible to ETEC and develop clinical diarrhea, they either exhibit species-specificity in bacterial attachment and intestinal colonization or are impractical due to limited availability and financial or ethical constraints [17,19].

Different approaches have been attempted to overcome the challenges in ETEC vaccine development. Developing a vaccine that covers all ETEC adhesins is currently not feasible. Thus, one strategy is to target a few adhesins that are associated with the majority of diarrheal clinical cases, especially the moderate-to-severe cases [39,40,41,42], or to identify conserved adhesins as vaccine antigens [43]. To overcome the challenge posed by STa’s potent toxicity and poor immunogenicity, genetic fusions or conjugations with STa peptides were attempted to generate safe antigens that induce neutralizing antibodies [39,40,41]. This review article outlines the application of novel epitope- and structure-based multiepitope-fusion-antigen (MEFA) for the creation of a polyvalent adhesin protein immunogen for broad immunity against the seven most prevalent and virulent ETEC adhesins. It also summarizes the generation of a toxoid fusion protein for neutralizing antibodies against both ETEC toxins (STa, LT) and the development of a broadly protective ETEC vaccine candidate. Additionally, this paper introduces an application of a combined animal challenge model. This dual-animal challenge model allows us to evaluate the preclinical efficacy of the ETEC vaccine candidate (MecVax) against ETEC intestinal colonization and ETEC toxin-mediated clinical diarrhea.

## 2. CFA/I/II/IV MEFA, a Broadly Immunogenic and Protective ETEC Adhesin Antigen Constructed with a Novel Epitope- and Structure-Based Multiepitope-Fusion-Antigen (MEFA) Platform

ETEC strains expressing adhesins CFA/I, CFA/II (CS1, CS2, CS3), or CFA/IV (CS4, CS5, CS6) cause about two-thirds of the ETEC-associated diarrheal cases and the moderate-to-severe cases [42,44,45,46]. These seven ETEC adhesins (CFA/I, CS1-CS6) become the primary antigen targets for immunity against ETEC bacterial attachment and colonization in the small intestines, the first line of defense against ETEC infection [19]. Unlike the whole-cell approach that mixes four inactivated strains expressing four adhesins (CFA/I, CS3, CS5, CS6) [47] or three live strains producing six adhesins (CFA/I, CS1, CS2, CS3, CS5, CS6) [48] for broad immunity, we applied an epitope- and structure-based vaccinology platform called multiepitope-fusion-antigen (MEFA) and constructed a polyvalent chimeric protein as an adhesin immunogen for broad immunity against the seven ETEC adhesins (CFA/I, CS1-CS6) [49].

**Figure 1:**
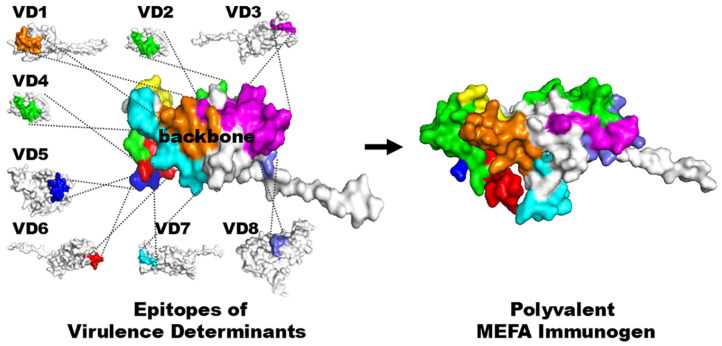
Scheme of the epitope- and structure-based multiepitope-fusion-antigen (MEFA) vaccinology platform in constructing a polyvalent immunogen for broad immunity and cross-protection against heterogeneous virulence factors or strains. (This figure was modified from Figure 1 of Microorganism 2023, 11(10):e11102473 [56]).

This MEFA platform combines the epitope vaccine concept and structural vaccine concept. MEFA presents multiple foreign epitopes (from heterogeneous virulence factors or pathogenic strains) on a backbone immunogen for a polyvalent chimeric immunogen and maintains foreign epitope native antigenic propensity for broadly protective immunity (Figure 1), thus overcoming the virulence or antigen heterogeneity challenge [17,18,50]. This platform uses structural and computational biology techniques to replace surface-exposed epitopes on a backbone with B-cell and T-cell epitopes from other virulence determinants or heterogeneous strains of interest, creating a broadly immunogenic polyvalent MEFA immunogen. A backbone (ideally also a virulence determinant of interest) typically has a stable structure and multiple well-separated epitopes, can be expressed using commonly used vector systems, and can be easily extracted with routine laboratory protocols. B-cell or T-cell epitopes can be predicted in silico using prediction software, including the IEDB (http://www.iedb.org), whereas functional or protective epitopes are identified empirically [51,52,53,54,55]. The position and presentation of the foreign epitopes on the MEFA are initially assessed and adjusted in silico to maintain native antigenic properties and then further evaluated empirically for broad immunogenicity and cross-protection against infections caused by heterogeneous strains [50].

**Figure 2:**
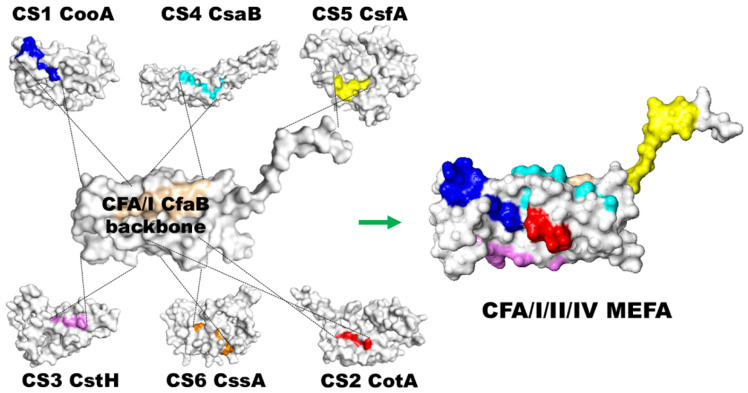
Illustration of the polyvalent ETEC adhesin immunogen, CFA/I/II/IV MEFA. Assisted by the MEFA platform, we presented the B-cell epitopes of the major subunits from seven ETEC adhesins (CFA/I, CS1-CS6) on the backbone CFA/I major subunit CfaB to generate a polyvalent protein immunogen CFA/I/II/IV MEFA.

Aided by this MEFA platform, we aimed to develop a broadly protective MEFA immunogen targeting the seven most significant ETEC adhesins (CFA/I, CS1-CS6). Using the major structural subunit of adhesin CFA/I, CfaB, as the backbone, we retained the most immunogenic B-cell conformational epitopes of the backbone, replaced the remaining epitopes with the most immunodominant epitope from each major structural subunit of the other six important ETEC adhesins, CooA of CS1, CotA or CS2, CstH of CS3, CsaB of CS4, CsfA of CS5, and CssA of CS6, and constructed a chimeric polyvalent protein, CFA/I/II/IV MEFA (Figure 2). This CFA/I/II/IV MEFA gene was synthesized and expressed in *E. coli* BL21 (DE3) [49], and recombinant CFA/I/II/IV MEFA protein had a stable tertiary structure close to backbone CfaB and the foreign epitopes retained native antigenic propensity [57].

This CFA/I/II/IV MEFA protein elicited robust antibody responses to all seven ETEC adhesins, and the antigen-specific antibodies blocked adherence of ETEC bacteria that produce any of the seven adhesins. Mice intraperitoneally immunized with the 6xHis-tagged CFA/I/II/IV MEFA protein, adjuvanted with incomplete Freund’s adjuvant (Sigma), or intramuscularly or subcutaneously administered with the tagless CFA/I/II/IV MEFA and adjuvanted with double mutant LT (dmLT; LT_R192G/L211A_), developed high titers of IgG antibodies to CFA/I, CS1, CS2, CS3, CS4, CS5, and CS6 [49,58,59]. Moreover, mouse serum antibodies significantly blocked the adherence of ETEC or *E. coli* strains expressing any of the seven target adhesins (blocking 40–75% of bacterial adherence to Caco-2 cells) [49,58,59].

More importantly, this CFA/I/II/IV MEFA protein antigen protected against ETEC bacterial intestinal colonization. Rabbits immunized intradermally or intramuscularly with CFA/I/II/IV MEFA protein, adjuvanted with 1 μg dmLT, developed robust IgG antibodies to the seven adhesins: CFA/I, CS1-CS6. Rabbit serum antibodies significantly blocked the in vitro adherence of ETEC or *E. coli* strains expressing CFA/I, CS1-CS6 adhesins, reducing bacterial adherence by 50–77% (CFUs). Furthermore, the immunized rabbits, when challenged with the ETEC strain B7A (CS6, STa, LT), exhibited a 2- to 3-log reduction in bacterial colonization of the small intestine. Protection against B7A bacterial intestinal colonization in the rabbits intradermally or intramuscularly with CFA/I/II/IV MEFA protein was the same as in the rabbits that were challenged (equivalent to oral immunization with B7A) and rechallenged (equivalent to oral challenge) with B7A. This indicated that parenterally administered CFA/I/II/IV MEFA protein antigen protects against ETEC colonization in the small intestines [60].

## 3. Toxoid Fusion 3xSTa_N12S_-mnLT_R192G/L211A_, a Nontoxic Toxin Antigen That Induces Neutralizing Antibodies Against ETEC Toxins STa and LT

ETEC adhesins mediate bacterial attachment to host cells and subsequent colonization of the host small intestine; anti-adhesin immunity serves as a first-line defense against ETEC infection. However, it is the enterotoxins produced by ETEC that directly disrupt homeostasis in intestinal epithelial cells, resulting in fluid hypersecretion and watery diarrhea. Thus, antitoxin immunity plays a crucial role in protecting against ETEC diarrhea. Additionally, toxin-mediated fluid secretion in epithelial cells disrupts epithelial tight junctions, further enhancing ETEC colonization in the small intestine [61]. Since ETEC strains producing STa or LT alone sufficiently cause diarrhea, an effective ETEC vaccine would need to carry both toxin antigens to stimulate protective antibodies against LT and STa. To achieve this goal, we need to address two key issues. The first is to eliminate the potent toxicity of LT and STa, allowing them to serve as safe antigens. The second is to facilitate the poor immunogenicity of STa, as this small-sized toxin naturally does not stimulate host immune response [19].

Past efforts to overcome ETEC toxin enterotoxicity have achieved limited success. For LT, the nontoxic B subunit, LTB, was used as a safe antigen to induce anti-LT antibodies. Antibodies to the LTB prevent the AB_5_ LT holotoxin from binding to the host receptor GM1, which is facilitated by the LTB pentamer, blocking LT endocytosis into host intestinal epithelial cells to some degree. However, anti-LTB antibodies do not neutralize LT enterotoxicity, which is contributed to by the A subunit (LTA) and plays a crucial role in causing water secretion from intestinal epithelial cells. The bigger problem lies in identifying safe antigens to induce neutralizing antibodies against the potent STa toxin. Early studies found that disruption of disulfide bonds, truncation, mutation, or fusion to a carrier abolished or reduced the biological toxicity of STa [62,63,64,65,66]. However, these modifications to the 19-amino acid peptide significantly alter the structural and antigenic properties of STa, and the resultant molecules (after being fused or coupled to a carrier) were unable to elicit antibodies against the native STa toxin. Indeed, it was speculated that only native STa or STa peptides with enterotoxicity can induce antibodies that react with STa and neutralize its enterotoxicity [67,68].

In a study of developing a vaccine against ETEC diarrhea in pigs, we noticed that a full-length pig ETEC-originated STa (pSTa or STp, an 18-amino acid peptide, homologous to the 19-amino acid human ETEC STa) abolished enterotoxicity after the substitution at the 11 12, or 13 residue. More profoundly, these full-length STa toxoids, after being genetically fused to the C-terminus of a monomeric LT mutant, showed no STa or LT enterotoxicity but induced antitoxin antibodies neutralizing both toxins [28]. This monomeric LT mutant had the 192 residue of the LTA subunit mutated and fused to the LTB subunit as an A_1_B_1_ monomer (not AB_5_ holotoxin). Pregnant pigs (sows) intramuscularly immunized with an STa-LT toxoid fusion developed neutralizing antibodies to both toxins. Furthermore, piglets born to the immunized mother were protected against clinical diarrhea after challenge with an STa ETEC strain [28]. One STa toxoid, even with the truncation of four N-terminal amino acids, elicited neutralizing anti-STa antibodies when this shortened STa toxoid peptide was fused to an adhesin major subunit gene and expressed in a chimeric adhesin [69].

Studies of the pig ETEC STa and LT toxoids and toxoid fusions highlighted that (1) mutations at a few non-cysteine residues of STa and fusion of a mutant LT A subunit with an LTB subunit deprive STa or LT of enterotoxicity, as they can no longer elevate intracellular cyclic guanosine or adenosine monophosphate or stimulate fluid secretion in gut loops, and (2) STa-LT toxoid fusions enhance STa immunogenicity and induce antibodies that neutralize STa and LT enterotoxicity and protect against clinical diarrhea. Encouraged by these exciting results, we expanded our investigation to study human ETEC STa and LT toxoids, as well as STa-LT toxoid fusions. We created a full-length STa toxoid STa_P13F_, which had the 13 residue proline replaced with phenylalanine. Then we genetically fused STa_P13F_ to the N-terminus, C-terminus, or between the A1 and A2 domains, or A and B subunit domains, of a monomeric LT toxoid, LT_R192G_, for STa-LT toxoid fusions. We found that serum antibodies from mice immunized with each of the four STa_P13F_-mnLT_R192G_ toxoid fusions neutralized STa toxin at levels that were either the same or similar [29].

**Figure 3:**
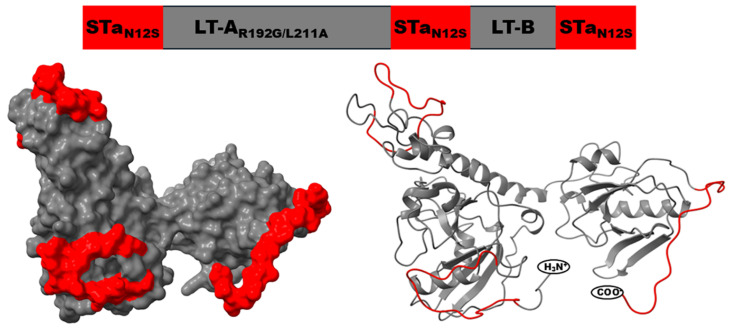
Illustration of the polyvalent ETEC toxoid fusion protein immunogen, 3xSTa_N12S_-mnLT_R192G/L211A_. By genetically fusing three copies of STa toxoid, STa_N12S_, to a monomeric LT double mutant, mnLT_R192G/L211A_, we created an STa-LT toxoid fusion immunogen. Note: STa toxoid is in rd.

Since an STa toxoid on either terminus or inside the monomeric LT_R192G_ maintained STa antigenicity similarly, we next fused three copies of an STa toxoid to a monomeric LT double mutant (mnLT_R192G/L211A_) on both termini, as well as between the A and the B subunit domains, to further enhance STa antigenicity (Figure 3). The initial study indicated that an STa-LT toxoid fusion carrying three copies of an STa toxoid increased anti-STa immunogenicity [70]. In a subsequent study, we constructed a panel of 12 6xHis-tagged 3xSTa-LT toxoid fusion proteins, each carrying a different STa toxoid, and compared their immunogenicity and antibody-neutralization activities [39]. While all 12 toxoid fusions were nontoxic and immunogenic, the toxoid fusion carrying STa toxoid STa_N12S_ (that has the 12th asparagine substituted by serine), 3xSTa_N12S_-dmLT, was the most effective in eliciting neutralizing anti-STa antibodies [39]. This 3xSTa_N12S_-dmLT was later named as 3xSTa_N12S_-mnLT_R192G/L211A_, to differentiate the A_1_B_1_ monomeric mnLT_R192G/L211A_ from the A_1_B_5_ holotoxin-structured LT_R192G/L211A_, dmLT. Serum antibodies from the mice parenterally administered with 3xSTa_N12S_-mnLT_R192G/L211A_, with a different adjuvant (Freund’s adjuvant, SEPPIC ISA51, or dmLT), neutralized STa and LT enterotoxicity [39,71]. More importantly, toxoid fusion 3xSTa_N12S_-mnLT_R192G/L211A_ induced antibodies that protected against STa ETEC clinical diarrhea. Piglets born to mothers intramuscularly immunized with this toxoid fusion protein, adjuvanted with dmLT, acquired maternal antitoxin antibodies; when challenged with an STa ETEC strain, these piglets were protected against clinical diarrhea [72].

We have overcome a long-standing obstacle in ETEC vaccine development by identifying a nontoxic ETEC toxin antigen that induces antibodies neutralizing STa and LT enterotoxicity and protecting against STa ETEC clinical diarrhea. But another concern was raised immediately: whether the protective anti-STa antibodies derived from a safe toxoid fusion antigen react with guanylin or uroguanylin. Guanylin and uroguanylin are the two STa-like ligands that critically regulate fluid and electrolyte transport and maintain homeostasis in human intestinal and renal epithelial cells. Cross-reactivity from anti-STa antibodies induced by ETEC vaccines with guanylin or uroguanylin would raise serious health concerns. To address this issue, we conducted a study to measure the cross-reactivity of anti-STa antibodies to the two ligands. We found that the neutralizing anti-STa antibodies derived from a toxoid fusion carrying an STa toxoid with a single mutation (STa_N12S_), a double mutation (STa_L9A/N12S_ or STa_N12S/A14T_), or a triple mutation (STa_L9A/N12S/A14T_) had little or no reactivity with guanylin or uroguanylin [73]. This paves the way for the use of toxoid fusion 3xSTa_N12S_-mnLT_R192G/L211A_ as a safe ETEC vaccine antigen.

## 4. MecVax, an ETEC Vaccine Candidate Composed of CFA/I/II/IV MEFA and Toxoid Fusion 3xSTa_N12S_-mnLT_R192G/L211A_, for Broad Protection Against ETEC Intestinal Colonization and Clinical Diarrhea

To develop an ETEC vaccine to induce broad anti-adhesin antibodies against ETEC bacterial intestinal colonization and also antitoxin antibodies against both STa and LT toxins, we combined the CFA/I/II/IV MEFA with the toxoid fusion 3xSTa_N12S_-mnLT_R192G/L211A_. CFA/I/II/IV MEFA was demonstrated to prevent in vitro adherence of ETEC bacteria expressing any of the seven important adhesins (CFA/I, CS1-CS6) and in vivo colonization in rabbit small intestines against ETEC strain B7A [49,59,60]. Toxoid fusion 3xSTa_N12S_-mnLT_R192G/L211A_ was shown to be nontoxic and induce antibodies to neutralize STa and LT enterotoxicity but also protect against STa ETEC clinical diarrhea in pigs [39,72]. These two protein antigens, however, were initially constructed to carry a 6xHis tag, which is considered undesirable for human vaccines. Additionally, it is untested whether these two proteins are antigenically compatible for co-administration. Therefore, we first constructed a tagless CFA/I/II/IV MEFA and a tagless toxoid fusion 3xSTa_N12S_-mnLT_R192G/L211A_ and verified each new antigen for broad immunogenicity. We then examined whether the tagless CFA/I/II/IV MEFA and the tagless toxoid fusion protein compromised each other’s antigenicity when administered together.

We found no differences in protein expression, immunogenicity, or antibody functions of the tagless and 6xHis-tagged CFA/I/II/IV MEFA or toxoid fusion 3xSTa_N12S_-mnLT_R192G/L211A_. Both 6xHis-tagged and tagless counterparts were expressed by *E. coli* BL21 (DE3) as inclusion body proteins and subsequently solubilized and refolded. They had the same level of yield, integrity or purity estimated based on SDS-PAGE Coomassie blue staining and reaction with antigen-specific antibodies [58]. Moreover, mice immunized with the tagless or 6xHis-tagged CFA/I/II/IV MEFA or toxoid fusion 3xSTa_N12S_-mnLT_R192G/L211A_ developed the same level of IgG antibodies to the seven ETEC adhesins (CFA/I, CS1-CS6) or toxins STa and LT. The derived antibodies in mouse sera exhibited the same levels of functional activity against adherence of ETEC bacteria expressing any of the seven target adhesins or against enterotoxicity induced by STa or CT (cholera toxin, LT homolog) [58].

We subsequently immunized mice intramuscularly or subcutaneously with the tagless CFA/I/II/IV MEFA, the tagless toxoid fusion 3xSTa_N12S_-mnLT_R192G/L211A_, or both. We observed that mice developed the same levels of anti-adhesin antibodies to the seven ETEC adhesins after immunization with the tagless CFA/I/II/IV MEFA alone or together with toxoid fusion 3xSTa_N12S_-mnLT_R192G/L211A_, or the same levels of anti-STa and anti-LT antibodies after immunization with toxoid fusion 3xSTa_N12S_-mnLT_R192G/L211A_ alone or in combination with CFA/I/II/IV MEFA [74,75]. Moreover, the derived mouse serum antitoxin antibodies, or anti-adhesin antibodies, from a single antigen or both proteins, equally inhibited ETEC bacterial adherence or neutralized STa or LT enterotoxicity [74,75]. Data from these studies demonstrated that the tagless toxoid fusion 3xSTa_N12S_-mnLT_R192G/L211A_ and CFA/I/II/IV MEFA retain broad immunogenicity and are antigenically compatible for co-administration.

**Figure 4:**
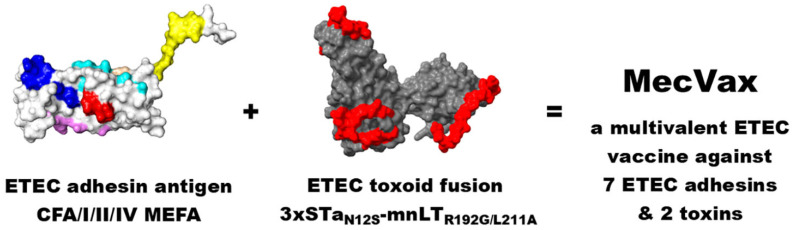
Scheme of MecVax, a protein-based multivalent ETEC vaccine candidate. MecVax is composed of two polyvalent proteins, CFA/I/II/IV MEFA and toxoid fusion 3xSTa_N12S_-mnLT_R192G/L211A_ to protect against the seven most significant ETEC adhesins (CFA/I, CS1-CS6) and two enterotoxins (STa and LT), which are associated with all ETEC clinical cases. Note: CFA adhesin epitopes and STa toxoid are indicated in different colors.

We developed MecVax, a multivalent ETEC vaccine candidate, by combining the tagless CFA/I/II/IV MEFA and toxoid fusion 3xSTa_N12S_-mnLT_R192g/L211A_ [58,74] (Figure 4). This vaccine candidate is to induce host anti-adhesin immunity to prevent adherence and intestinal colonization from any ETEC strains with any of the seven adhesins (CFA/I, CS1-CS6; together with STa and/or LT toxin). This vaccine is also to induce antitoxin immunity to neutralize the enterotoxicity of ETEC toxins STa and LT, which, alone or together, are produced by all ETEC strains. Therefore, the anti-adhesin antibodies induced by MecVax protect against infection from ETEC strains that express the seven adhesins, and the antitoxin antibodies protect against ETEC strains that express STa and/or LT toxin together with the seven adhesins or any other adhesins, thus protecting against all ETEC infections and associated diarrhea.

MecVax demonstrated broad immunogenicity and cross-protective activity against ETEC intestinal colonization and clinical diarrhea in preclinical studies [74,75,76,77,78,79,80]. MecVax, administered intramuscularly or intradermally, elicited robust antibody responses to the seven adhesins and two toxins in mice [74,75], even at a dose as low as 3 μg per protein antigen [76]. MecVax-induced mouse serum antibodies, just as the antibodies derived from the CFA/I/II/IV MEFA or the toxoid fusion, prevented the adherence of ETEC strains expressing any of the seven adhesins (CFA/I, CS1-CS6) by 40% to 66% and completely neutralized the enterotoxicity of STa and LT (CT homolog). When administered intramuscularly to pregnant sows, adjuvanted with dmLT, MecVax elicited robust serum and colostrum IgG and colostrum IgA to the seven target adhesins (CFA/I, CS1-CS6) and two toxins (STa, LT), and the born piglets acquired antigen-specific maternal antibodies (serum IgG). Sow serum or colostrum antibodies, as well as piglet serum antibodies, inhibited adherence of ETEC strains expressing the target adhesins and neutralized STa and LT enterotoxicity. Moreover, after challenge with an STa or an LT ETEC strain, piglets born to immunized mothers were protected at 100% against watery diarrhea and 71% or 92% against any diarrhea [74].

Adult rabbits intramuscularly immunized with MecVax developed antigen-specific IgG antibodies. Rabbit serum antibodies prevented 51% to 66% ETEC bacterial adherence to Caco-2 cells. When orally infected with the ETEC strain H10407 (CFA/I, STa, LT), the immunized rabbits showed an over 99.9% reduction in H10407 colonization in the small intestine compared to the control rabbits [77]. Similarly, when challenged with ETEC field isolate EL392-75 (CS1/CS3, STa, LT), ETP05011 (CS2/CS3, STa, LT), E106 (CS4/CS6, STa, LT), UM75688 (CS5/CS6, STa, LT), or B7A (CS6, STa, LT), the rabbits intramuscularly immunized with MecVax were protected from 99% to 99.9% bacterial colonization in small intestines [78].

Data from the rabbit and pig immunization and challenge studies clearly demonstrated that MecVax is broadly immunogenic and protective. This protein-based injectable ETEC vaccine candidate protected against ETEC intestinal colonization and clinical diarrhea. MecVax can induce robust IgG and IgA (in pregnant sows) antibodies to the seven ETEC adhesins (CFA/I, CS1-CS6) and two toxins (STa, LT). The vaccine-induced antibodies are broadly functional, inhibiting adherence of ETEC strains that produce any of the seven adhesins and neutralizing both toxins. Furthermore, MecVax, administered intramuscularly, prevents ETEC colonization of the rabbit intestine and protects newly born piglets that acquired maternal antibodies from the immunized sows against ETEC clinical diarrhea. It is worth noting that our rabbit immunization and challenge studies showed intramuscular immunization with a protein-based vaccine adjuvanted with dmLT protects against colonization of the small intestine by enteric bacteria such as ETEC or *Vibrio cholerae* [60,74,77,78,81]. This certainly warrants further investigation into how, and to what extent, the primarily systemic anti-adhesin (and antitoxin) immunity provides local mucosal protection against intestinal colonization by ETEC or other enteric pathogens.

Additionally, MecVax, when supplemented with another polyvalent protein immunogen, broadens protection against additional ETEC strains or other enteric pathogens. When co-administered with CFA MEFA-II, another polyvalent ETEC adhesin immunogen that covers five second-tier ETEC adhesins (CS7, CS12, CS14, CS17, CS21) [82], MecVax induced anti-adhesin antibodies to inhibit adherence from ETEC bacteria expressing any of the twelve target ETEC adhesins (CFA/I, CS1-CS7, CS12, CS14, CS17, CS21), as well as to neutralize STa and LT enterotoxicity, potentially preventing adherence and intestinal colonization from the ETEC adhesins that are associated with 86% of ETEC diarrheal clinical cases and nearly all moderate-to-severe cases [83].

Similarly, after co-administration with a polyvalent *Shigella* MEFA protein, intramuscularly immunized MecVax (and the *Shigella* MEFA) induced IgG antibodies to the nine ETEC antigens (CFA/I, CS1-CS6, STa, LT) and seven *Shigella* antigens (IpaB, IpaD, GuaB, VirG, StxA, Stx2A, StxB). This *Shigella* MEFA presents heterogeneous epitopes of *Shigella* invasion plasmid antigen B (IpaB) and D (IpaD), virulence factor G (VirG), and Shiga toxins (Stx, Stx2) on backbone IpaD and is designed to provide broad protection against shigellosis [84]. These antibodies inhibited the adherence of ETEC bacteria producing any of the seven adhesins, neutralized ETEC STa and LT toxicity, inhibited the adherence of all four *Shigella* species (*S. flexneri*, *S. sonnei*, *S. boydii*, and *S. dysenteriae*) and the important serogroup strains, blocked the invasion of *Shigella* strains, and neutralized the cytotoxicity of Shiga toxins. Most interestingly, co-administration of MecVax and *Shigella* MEFA protected against *Shigella* lethal pulmonary infections, ETEC bacterial intestinal colonization, and ETEC clinical diarrhea, leading to the development of a combined vaccine candidate, ShecVax, to protect against ETEC and *Shigella* infections [85].

While MecVax clearly shows potential as a broadly protective vaccine against ETEC diarrhea and as a combined vaccine against ETEC and other enteric bacteria, its clinical efficacy has yet to be investigated. Currently, efforts are underway to optimize MecVax processing and analytical development toward GMP vaccine production, with specific attention to downstream processing to improve product purity and uniformity further and to scale up production. Afterwards, MecVax will be evaluated for safety and broad immunogenicity in human volunteers, then efficacy in the controlled human infection model (CHIM) and field trials.

## 5. MecVax Preclinical Efficacy Can Be Evaluated in a Dual Animal Challenge Model, a Combination of a Rabbit Model Against ETEC Intestinal Colonization and a Pig Passive Protection Model Against ETEC Toxin-Mediated Diarrhea

Animal models for testing vaccine immunogenicity, safety, and efficacy are a cornerstone of vaccine development and are invaluable. A suitable animal model uses an animal species that is naturally susceptible to the pathogen, develops identical or similar clinical outcomes to humans after infection, tricks the immune response, and remains protected against subsequent homologous infection. Unfortunately, there is no suitable animal model for preclinical evaluation of vaccine efficacy in ETEC. The species commonly used in research laboratories, including rodents, are not naturally susceptible to ETEC or do not develop diarrhea after infection; thus, they provide limited value in ETEC vaccine research and development [17,19]. The nonhuman primate *Aotus nancymaae*, on the other hand, is susceptible to ETEC, develops diarrhea after challenge, and can serve as a good infection model to test ETEC vaccine candidacy [86]. However, resource scarcity, high costs, and ethical concerns make this model essentially unattainable for most research laboratories.

Rabbits, particularly under the RITARD (removable intestinal tie adult rabbit diarrhea) model, can be colonized by ETEC or *Vibrio cholerae* in the small intestine, enabling them to be used to study ETEC pathogenesis and protection of anti-adhesin immunity [33,87]. However, although ETEC bacteria can colonize the rabbit’s small intestine after oral inoculation, infected rabbits rarely develop clinical diarrhea. Therefore, rabbits can be used to evaluate ETEC vaccine candidates for efficacy against ETEC intestinal colonization but not against ETEC-associated clinical diarrhea.

On the other hand, pigs, particularly young pigs, are naturally susceptible to ETEC and develop clinical diarrhea, as well as anti-adhesin and antitoxin antibodies, after infection. However, the ETEC strains that cause diarrhea in pigs produce host-specific fimbrial adhesins to recognize pig-specific receptors in the pig small intestine. Adhesins of human ETEC strains do not attach to pig receptors and thus cannot colonize the pig small intestine. Therefore, pigs cannot be used to evaluate the protection conferred by ETEC vaccine candidates against intestinal colonization by human ETEC strains. On the other hand, STa and LT toxins produced by pig- or human-specific ETEC bacteria are highly homologous [36]. Indeed, recombinant *E. coli* strains expressing a pig-specific adhesin and the pig-type or human-type STa or LT equally elevate intracellular cGMP or cAMP and stimulate fluid accumulation in the ligated small intestinal loops in pigs [88]. Pigs orally inoculated with an ETEC strain expressing a pig-type adhesin and a human ETEC STa or LT toxin develop watery diarrhea [41,72,89]. That makes pigs a challenging model for assessing vaccine efficacy against ETEC toxin-mediated clinical diarrhea [19,41,72,90].

Now that a rabbit model can be used to evaluate ETEC vaccine candidate protection against ETEC bacterial intestinal colonization but not against clinical diarrhea, whereas a pig model can assess vaccine efficacy against toxin-mediated clinical diarrhea but not against ETEC intestinal colonization, a dual-animal challenge model that combines a rabbit colonization model and a pig infection model enables us to examine protection against bacterial intestinal colonization and clinical diarrhea synergistically, making it suitable to evaluate ETEC vaccine candidacy.

Examined with a rabbit colonization model, MecVax prevented ETEC colonization in rabbit small intestines significantly, showing a two- to three-log (99–99.9%) reduction by ETEC wildtype strains expressing CFA/I, CS1/CS3, CS2/CS3, CS3, CS4/CS6, CS5/CS6, or CS6 adhesins [77,78]. When evaluated in a pig passive infection model, MecVax protected piglets born to mothers immunized with MecVax from watery diarrhea (100%) and any diarrhea (71–92%) when challenged with recombinant ETEC strains with a pig-specific adhesin (p87P) and a human-type STa or LT toxin [74,85]. Combining the preclinical efficacy data from the dual-animal challenge model, we conclude that MecVax is broadly effective against ETEC intestinal colonization and clinical diarrhea, potentially serving as a broadly protective vaccine against ETEC-associated children’s diarrhea and travelers’ diarrhea.

## 6. Conclusions

An effective vaccine is urgently needed to protect against ETEC-caused diarrhea in children and travelers. Progress has been made in developing broadly protective ETEC vaccines, including three advanced to Phase 2 studies [17]. MecVax, a protein-based multivalent ETEC vaccine candidate composed of two polyvalent proteins, unprecedentedly targets seven ETEC adhesins (CFA/I, CS1-CS6), which are associated with two-thirds of clinical cases, and both toxins (STa, LT), which cause all clinical cases. MecVax induces functional antibodies against the adherence of ETEC bacteria expressing any of the seven adhesins and the enterotoxicity of both ETEC toxins and also prevents ETEC bacterial intestinal colonization and toxin-mediated clinical diarrhea in a rabbit-pig dual-animal challenge model. Future studies examining efficacy in clinical trials will determine whether MecVax is an effective vaccine against ETEC-associated children’s diarrhea and travelers’ diarrhea.

## Data Availability

All data were published in the original publications and are available.

## Abbreviations

The following abbreviations are used in this manuscript:

ETEC	Enterotoxigenic *Escherichia coli*
MEFA	Multiepitope fusion antigen
MecVax	Multivalent ETEC vaccine
CFA	Colonization factor antigen
CS	Coli surface antigen
LT	Heat-labile toxin
STa	Heat-stable toxin
WASH	Water, sanitation, hygiene
AMR	Antimicrobial resistance
cAMP	Cyclic adenosine monophosphate
cGMP	Cyclic guanosine monophosphate
LTA	Heat-labile toxin A subunit
LTB	Heat-labile toxin B subunit
GM1	GM1 gangliosidosis
dmLT	Double mutant heat-labile toxin
IgG	Immunoglobin G
IgA	Immunoglobin A
CHIM	Controlled human infection models
